# Multimodal locking-enabled robust and day-scale low-repetition-rate soliton microcomb for high-precision metrology

**DOI:** 10.1038/s41377-026-02460-w

**Published:** 2026-09-07

**Authors:** Jian Tang, Jun Yang, Enqi Yan, Guangyao Huang, Ke Yin, Donglai Tian, Xiaoyang Lei, Jiyuan Huang, Mingyue Yang, Haoran Ding, Shuhua Yan, Ke Wei, Lingxiao Zhu, Guochao Wang, Tian Jiang

**Affiliations:** 1 College of Intelligence Science and Technology, National University of Defense Technology, 410073 Changsha, China; 2 National Key Laboratory of Equipment State Sensing and Smart Support, National University of Defense Technology, 410073 Changsha, China; 3 College of Advanced Interdisciplinary Studies, National University of Defense Technology, 410073 Changsha, China; 4 College of Science, National University of Defense Technology, 410073 Changsha, China

**Keywords:** Frequency combs, Integrated optics

## Abstract

Chip-scale soliton microcombs, particularly those operating at low, electronically detectable repetition rates (≤ 26.5 GHz), are highly promising for portable metrology. However, their practical deployment has been critically hindered by poor robustness against intracavity noise and environmental perturbations. Here, we overcome this limitation by proposing a multimodal locking architecture that actively and simultaneously stabilizes all three fundamental parameters in a soliton microcomb: the pump frequency, the cavity resonance, and the repetition rate. This architecture is confirmed both theoretically and experimentally. Implemented on a Si_3_N_4_ microresonator with an FSR of 24.96 GHz, this approach enables robust soliton generation and sustains record-long, collapse-free operation for over 48 h. More importantly, it maintains soliton robustness with exceptional resilience to environmental shocks, under temperature variations exceeding 10°C and vibration accelerations beyond ±4 g. The microcomb’s metrological utility as a precise optical ruler is further validated through optical frequency calibration and frequency-sweeping-based absolute ranging demonstration. This work provides a critical solution for robust and field-deployable low-repetition-rate soliton microcombs, paving the way for their use in portable optical clocks, high-precision ranging, time-frequency transfer and spectroscopic sensing.

## Abstract (web summary)

Implemented on a Si₃N₄ microresonator, a multimodal locking architecture simultaneously stabilizes pump frequency, cavity resonance, and repetition rate, enabling robust and day-scale low-repetition-rate soliton microcombs for precision metrology.

## Graphical abstract

**Figure:**
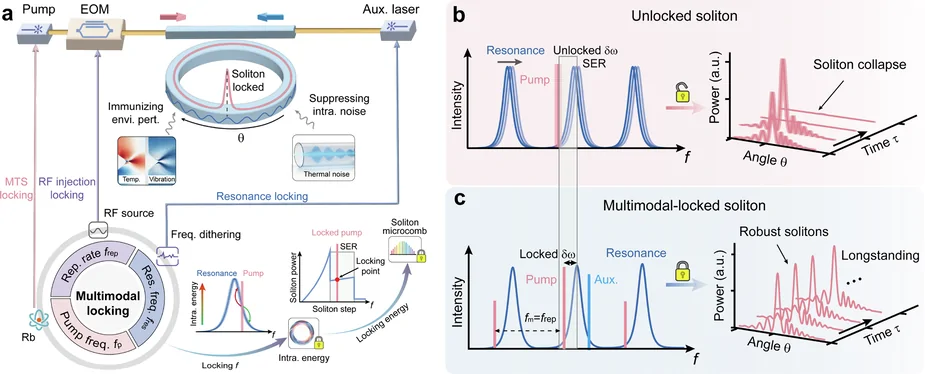

## Introduction

**Fig. 1:**
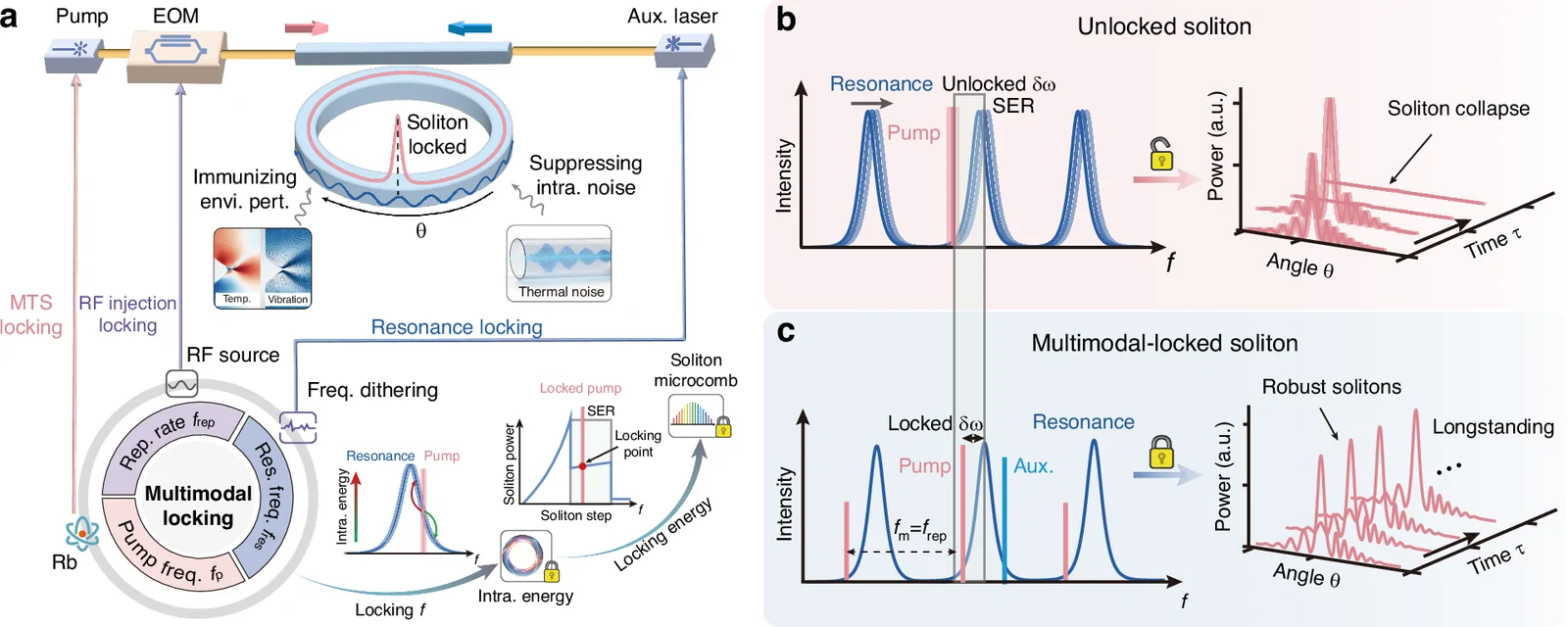
**Multimodal-locked soliton microcomb work concept**. **a** Schematic of the multimodal locking architecture. The intracavity energy is disturbed by environmental perturbations and intracavity noise, which in turn shifts the pump-cavity detuning, leading to soliton collapse. To enhance the soliton robustness, the pump laser is stabilized to a Rb reference via modulation transfer spectroscopy (MTS), providing an absolute frequency anchor. A counter-propagating auxiliary laser, blue-detuned to the same resonance, thermally broadens the soliton existence range (SER, shaded area). Its frequency is feedback-controlled via the detected soliton power to lock the pump-cavity detuning *δω* within the SER. The SER defines the limited maximum range of pump-cavity detuning *δω* where solitons can exist. Within this range, nonlinearity balances dispersion and parametric gain balances loss, forming the characteristic soliton step (shaded areas). Simultaneously, the repetition rate is phase-locked to an external Rb-clock-referenced RF source through microwave injection locking via an electro-optic modulator (EOM). Together, these three active locks stabilize the pump frequency, cavity resonance, and repetition rate, achieving full frequency and energy locking for robust long-term soliton operation. **b** Unlocked operation: Environmental perturbations shift the cavity resonance and pump frequencies. This alters *δω*, which disturbs the soliton power and repetition rate. Ultimately, *δω* is driven outside the SER, resulting in soliton collapse. **c** Multimodal-locked operation: active stabilization of the pump frequency, cavity resonance, and repetition rate maintains the soliton state robustly within the SER, enabling long-term stable operation

Optical frequency combs, serving as precise spectral rulers across the electromagnetic spectrum, have revolutionized precision metrology and fundamental science^1–4^. Their miniaturization through on-chip soliton microcombs^5–15^ represents a pivotal advance towards chip-scale integration, opening avenues for applications that demand high precision in compact formats, such as optical atomic clocks^16,17^, microwave signal synthesis^18–20^, laser ranging^21–24^, and precision spectroscopy^25,26^. Among these, soliton microcombs with electronically detectable low repetition rates (≤ 26.5 GHz, the typical electronic bandwidth of major instruments) hold particular value for field-deployable applications^27–29^. This intrinsic compatibility with microwave photonic architectures makes them ideal candidates for applications such as integrated spectroscopic sensors, next-generation coherent communication systems, and calibration of astronomical spectrographs (10–30 GHz)^30,31^, where the optical comb can be seamlessly mapped into the radio-frequency domain. However, the practical deployment of low-repetition-rate soliton microcombs is fundamentally constrained by two factors, namely the intracavity noise and environmental perturbations, which result in poor robustness (long-term and short-term stability as well as dependability). Specifically, on one hand, the intracavity noise is caused by a larger cavity volume (a smaller free spectral range), which translates even minor thermal noise into major resonance drift. Such resonance drift is further amplified by the narrow soliton existence range (SER)^32^ and the strong thermo-optic effect. Thus, it is particularly difficult to achieve dependability within this narrow SER and thereby compromising both long-term and short-term stability, such as frequency stability and phase noise (Supplementary Note 1). On the other hand, environmental perturbations, such as temperature fluctuations and mechanical vibrations, can readily disrupt the thermal equilibrium, leading to soliton collapse. Together, the combination of intracavity noise and environmental perturbations makes it difficult to sustain soliton states within the narrow SER, ultimately hindering low-repetition-rate soliton operation from leaving the laboratory environment (Fig. 1b).

Various technologies, such as frequency sweeping^14,32,33^, self-injection locking^34–36^, and auxiliary laser heating^37–39^, have been developed to initiate and sustain soliton states, which require the coordinated control of multiple parameters in the SER. Nevertheless, existing solutions for robust low-repetition-rate solitons remain partial and deficient in field-ready scenarios. Frequency sweeping overcomes nonlinear thermal hysteresis during soliton formation but operates as an open-loop process, offering no continuous correction for subsequent environmental drift. Self-injection locking leverages mutual coupling between the laser and the microcavity to enable passive tracking of the pump frequency with cavity mode drifts, thus extending the soliton lifetime to some extent. However, this method does not contribute to long-term or short-term stability. In contrast, the auxiliary laser heating technique offers enhanced control by injecting a blue-detuned auxiliary beam to compensate for intracavity thermal effects, thereby widening the deterministic SER. Although the above methods maintain thermal equilibrium to some extent, none of them offer long-term or short-term stability. To further improve stability, a single comb line (e.g., the pump laser) can be stabilized to an atomic reference^40,41^ to provide an absolute frequency anchor, but this approach controls only one degree of freedom. Furthermore, while injection locking (pump modulation)^42,43^ and phase locking^44^ for stabilizing the repetition rate of soliton microcombs offer inherent advantages due to electronic compatibility, these methods are also limited to a single dimension. Overall, the cavity resonance remains free to drift with temperature and vibration, leaving the soliton uncontrolled and fragile^45–47^. The soliton existence depends on the critical condition of intracavity energy balance, where the dynamical equilibrium is maintained between Kerr nonlinearity and dispersion. Such energy balance is determined by the pump laser frequency and resonance frequency^12,48^. Even a minute laser frequency drift can perturb the Kerr nonlinearity and disrupt the intracavity energy balance, leading to soliton collapse (Fig. 1a). Accordingly, active frequency control offers a clear route to stabilize intracavity energy for thermal equilibrium and dramatically enhance soliton robustness. Therefore, to achieve a truly robust low-repetition-rate soliton microcomb, it is necessary to multidimensionally stabilize interdependent frequencies.

Here, we propose a multimodal locking architecture that, for the first time, establishes full active stabilization of all three critical frequencies for a 24.96-GHz-repetition-rate Si_3_N_4_ soliton microcomb, yielding unprecedented long-term robustness (Fig. 1c). We also develop an extended Adler equation to describe the principle of multimodal locking. Our architecture incorporates a triple-locking strategy via the locking of the pump laser frequency, the cavity resonance frequency, and the repetition rate (Fig. 1). With this multimodal locking architecture, we achieve robust, frequency-stabilized, and collapse-free soliton operation for over 48 h—a milestone for sub-26.5 GHz devices. The system’s robustness is rigorously validated under environmental shocks, maintaining stability through temperature variations exceeding 10°C and mechanical vibrations accelerating beyond ±4 g. The comb’s metrological utility is also confirmed through application demonstrations, including optical frequency calibration and frequency-swept interferometry (FSI) ranging. Our work provides a comprehensive solution to the stability challenge, significantly advancing the prospects for deploying low-repetition-rate soliton microcombs in practical portable and field-deployable systems.

## Results

### Multimodal-locked single-soliton generation and long-term frequency stabilization

**Fig. 2:**
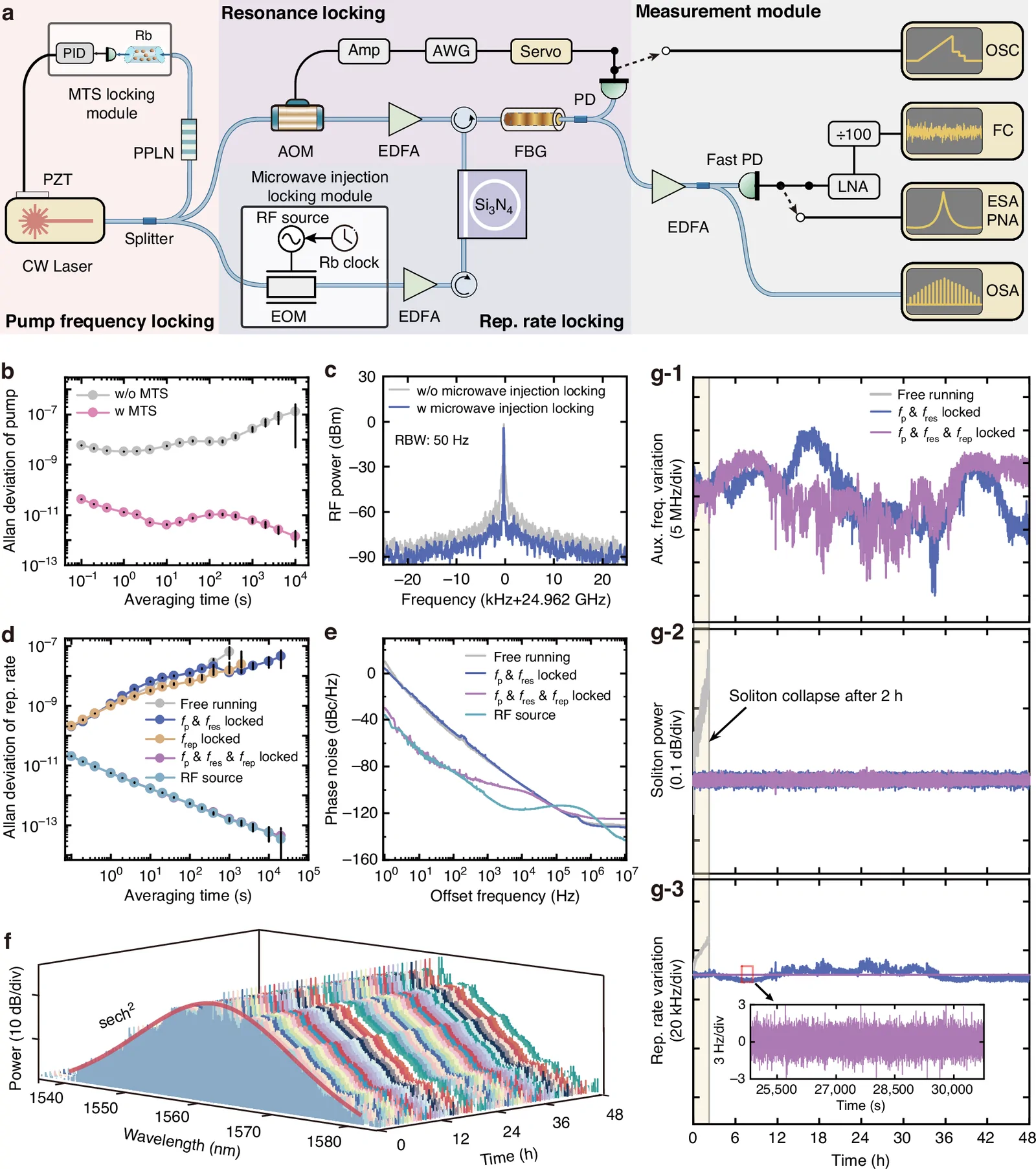
**Experimental setup and results of the multimodal-locked soliton microcomb**. **a** Experimental setup. Key components include: PZT, piezoelectric ceramic transducer; AOM, acousto-optic modulator; EOM, electro-optic modulator; AWG, arbitrary waveform generator; EDFA, erbium-doped fiber amplifier; FBG, fiber Bragg grating; PD, photodetector; LNA, low-noise amplifier; OSA, optical spectrum analyzer; OSC, oscilloscope; ESA, electrical spectrum analyzer; PNA, phase noise analyzer; FC, frequency counter. **b** Allan deviation of the pump laser before (gray) and after (red) locking to a Rb reference via modulation-transfer spectroscopy (MTS). **c** Electrical spectrum of the repetition rate without (gray) and with (blue) microwave injection locking. RBW, resolution bandwidth. **d** Allan deviation of the repetition rate. When locked, the stability reaches the level of the Rb‑clock‑referenced RF source, improving from 1.2 × 10^−9^ to 5.6 × 10^−12^ at 1 s, and from 6.4 × 10^−8^ to 5.8 × 10^−14^ at 10000 s. **e** Phase‑noise comparison. The locked repetition rate closely follows the external RF reference at low offset frequencies. **f** Time-lapse optical spectra recorded at hourly intervals over 48 h. **g** Long-term traces of (g-1) auxiliary-laser frequency, (g-2) soliton output power, and (g-3) repetition rate over 48 h. Inset in g‑3 shows the multimodal-locked repetition‑rate, fluctuating within ±3 Hz. The unchanged smooth sech^2^ envelope, along with continuous power and repetition rate records confirms the single-soliton operation during the 48-hour test

The stabilized soliton microcomb is implemented in a high-quality Si_3_N_4_ microring resonator with a free spectral range (FSR) of 24.96 GHz and a loaded quality factor of 4.4 × 10^6^ (Fig. 2a; see Supplementary Note 3 for characterization). We first deterministically access a single-soliton state using the auxiliary-laser heating technique. A counter-propagating, blue-detuned auxiliary laser is injected into the same resonance mode to manage nonlinear thermal dynamics during the initiation phase. By optimizing the pump power (200 mW), auxiliary power (195 mW), and the frequency shift applied to the auxiliary beam via an acousto-optic modulator (140 MHz), we effectively broaden the SER and facilitate reliable entry into the soliton regime (Supplementary Note 5 and Figs. S9, S10). Building on this initial state, we implement a multimodal locking architecture that actively controls the three fundamental parameters of the comb system: *f*_p_, *f*_res_, and *f*_rep_, as illustrated in Fig. 2a. Each lock operates via a dedicated active stabilization, collectively forming a robust defense against environmental perturbations.

To anchor the entire comb spectrum to an absolute frequency reference, the pump laser itself (*f*_p_) is first stabilized (Fig. 2a, red area). A portion of the 1560 nm pump light is frequency-doubled and locked to the ^87^Rb D_2_ transition using MTS (see setup in Supplementary Note 4). The derived error signal is fed back to the pump laser’s piezoelectric transducer, suppressing its long-term drift. The stability of this lock is quantified by beating the stabilized pump against a commercial Menlo optical frequency comb (OFC, FC1500-250-ULN). The Allan deviation of the beat note, shown in Fig. 2b, improves from 3.4 × 10^−9^ to 1.3 × 10^−11^ at 1 s averaging time and from 1.3 × 10^−7^ to 1.4 × 10^−12^ at 10000 s averaging time. This represents a greater than hundredfold reduction in short-term noise and an improvement by over four orders of magnitude in long-term stability. Fixing the pump laser frequency decouples it from *f*_res_, thereby reducing pump perturbations to the microcavity and relaxing the auxiliary-laser tuning range.

Maintaining the critical pump-cavity detuning *δω* (and thereby the *f*_res_) precisely within the SER (~ 45.5 MHz for our device) is crucial for soliton survival. We implement a real-time feedback loop to actively stabilize this parameter (Fig. 2a, purple area). The underlying principle is that the average power of the generated soliton *P*_sol_ scales with $\sqrt{\delta \omega }$^49^. Therefore, after filtering out the pump light with a fiber Bragg grating (FBG), we monitor *P*_sol_ and use it to obtain an error signal. A servo controller processes this signal and dynamically adjusts the frequency of the counter-propagating auxiliary laser. Changing the auxiliary laser frequency alters the total intracavity power, which via the thermo-optic effect shifts the cavity resonance, thereby dragging it to maintain *δω* at the desired setpoint. Combining this active *δω* lock with the above *f*_p_ lock yields a stable soliton whose power fluctuates within only ±0.01 dB over 48 h (Fig. 2g-2). During this period, the auxiliary laser frequency varies by up to ±10 MHz to compensate for thermal fluctuations (Fig. 2g-1), a range significantly less than the SER itself, underscoring the necessity of active stabilization to prevent collapse.

**Fig. 3:**
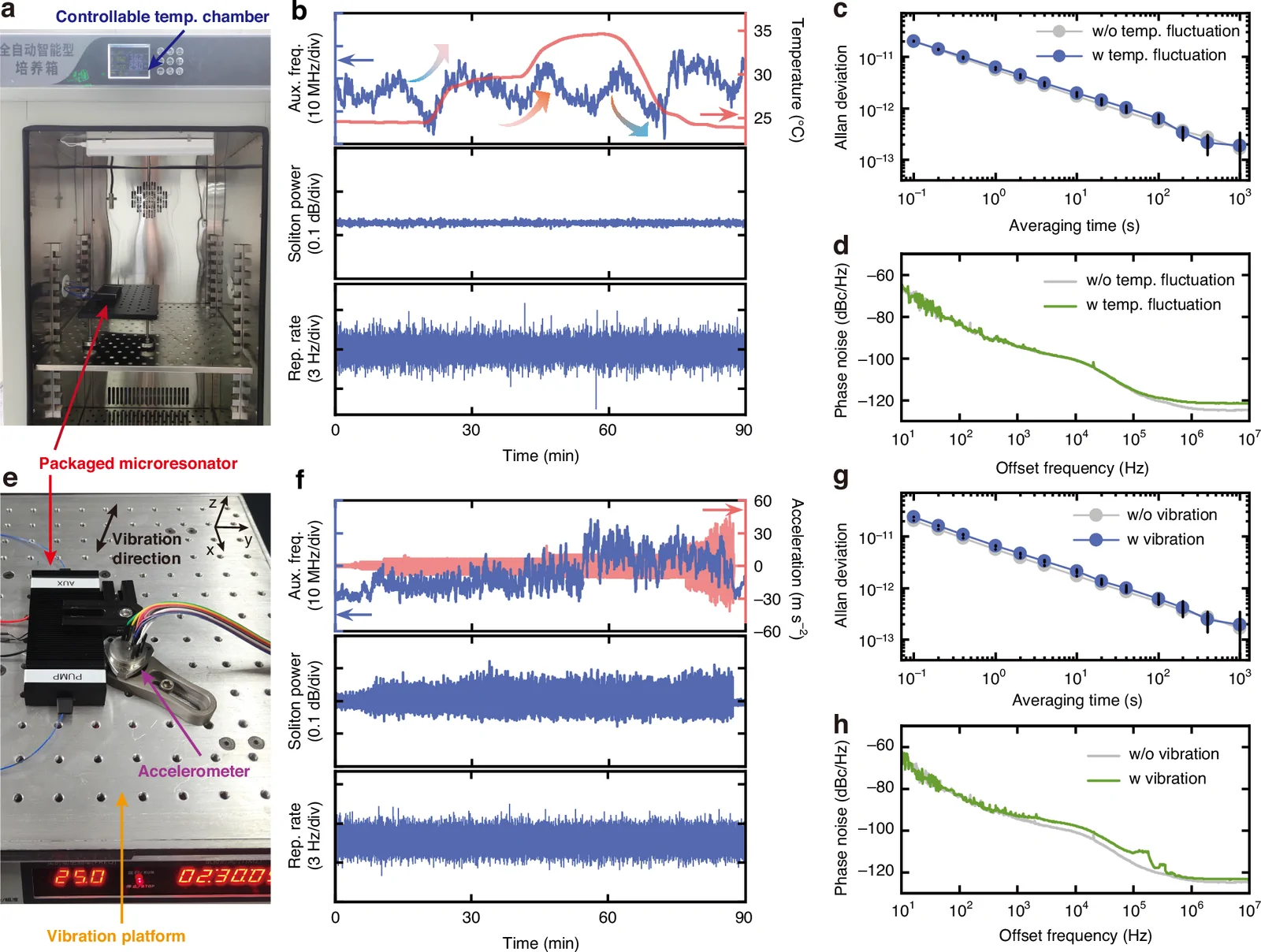
**Environmental robustness of the multimodal-locked single soliton**. **a** Photograph of the temperature-perturbation setup. **b** Recorded variations of ambient temperature, auxiliary laser frequency, soliton output power, and repetition rate during a 90‑min test with temperature swings from 23.9 °C to 34.7 °C. **c** Allan deviation of the repetition rate with and without temperature perturbation, showing minimal degradation. **d** Phase noise of the repetition rate with and without temperature fluctuation, showing consistency under temperature fluctuations. **e** Photograph of the vibration-test platform. The platform vibrates vertically at 25 Hz, with a vibration amplitude exceeding ±4 g. **f** Measured vibration acceleration (exceeding ±4 g), auxiliary laser frequency, soliton power, and repetition rate under 25 Hz vertical vibration. **g** Allan deviation of the repetition rate with and without mechanical vibration, demonstrating that the frequency stability is preserved under dynamic mechanical stress. **h** Phase noise of the repetition rate with and without mechanical vibration

Although the combined locking of *f*_p_ and *δω* enables a soliton with stable average power, the repetition rate *f*_rep_ still fluctuates ~ 15 kHz (Fig. 2g-3), mainly attributed to intracavity optical power variations induced by the frequency and power jitter of the pump and auxiliary lasers. Such a large *f*_rep_ fluctuation is unacceptable for precision applications. To further stabilize *f*_rep_, we employ microwave injection locking (Fig. 2a, blue area). An external radio-frequency source (Rohde & Schwarz SMA100B), itself referenced to the same rubidium clock, generates a signal at 24.962 GHz. This signal drives an electro-optic phase modulator placed on the pump path, imposing weak sidebands with modulation depth *β*_m_ = 0.08 on the pump laser. Its 24.3 kHz locking range is sufficient to cover the repetition rate fluctuation (Supplementary Note 2). When the RF frequency is tuned to match the microresonator’s FSR, the soliton pulse train synchronizes to this stable external reference. The electrical spectrum of the detected photocurrent, shown in Fig. 2c, demonstrates complete suppression of the unlocked, broadened repetition-rate peak, leaving a clean, narrow signal. The stability transfer is quantified in Fig. 2d-e. The *f*_rep_ Allan deviation (Fig. 2d) improves from 1.2 × 10^−9^ to 5.6 × 10^−12^ at 1 s and from 6.4 × 10^-8^ to 1.5 × 10^−13^ at 1000 s. For comparison, we measured the *f*_rep_ Allan deviation under microwave injection locking alone. Note that the long-term stability of *f*_rep_ is not improved, as cavity drift caused by environmental fluctuations pushes it outside the limited locking range (see Supplementary Note 2 for details). This effectively confirms the significant contribution of multimodal locking. The *f*_rep_ phase-noise (Fig. 2e) closely follows the RF source for offset frequencies below 300 Hz, achieving ~ 40 dB noise suppression. More importantly, the locking method effectively suppresses noise caused by backscattering from the auxiliary comb at high auxiliary laser powers (195 mW). For offset frequencies beyond 30 kHz, however, the phase noise gradually returns to the intrinsic noise floor of the free-running microcomb. This occurs because microwave injection locking primarily suppresses low-frequency noise (e.g., slow thermal drift) but is ineffective at higher offset frequencies, where the microresonator’s intrinsic noise dominates. These experimental findings are also confirmed by our newly generalized Adler equation based on the Lugiato-Lefever equation (see Materials and Methods and Supplementary Note 2).

The synergistic operation of the multimodal locking enables record-long, collapse-free operation of a low-repetition-rate single-soliton microcomb. The single-soliton state is identified by the smooth sech^2^ spectral envelope and the absence of multi-soliton interference spectra. The optical spectra, recorded at hourly intervals over 48 h, maintain a clean sech^2^ envelope without observable spectral modulation associated with multi-soliton states, breathing-induced spectral oscillation, or soliton collapse (Fig. 2f). Moreover, the residual fluctuation of *f*_rep_ is reduced to within ±3 Hz under full injection locking (inset of Fig. 2g-3). These results confirm that the soliton number remains unchanged at one throughout the 48-hour test. Thus, the multimodal-locking architecture transforms the chip-based microcomb into a frequency-stabilized optical frequency ruler, providing the long-term reliability and precision required for the metrological applications.

### Environmental robustness of the multimodal-locked single soliton

The robustness of the multimodal-locked soliton against environmental perturbations, a critical requirement for field deployment, is systematically evaluated under controlled temperature fluctuations and mechanical vibrations. The pump laser and auxiliary laser utilize an optimal power combination of 200 mW and 195 mW, respectively (Supplementary Note 5). To assess thermal robustness, the packaged microresonator is first placed inside a temperature-controlled chamber. As illustrated in Fig. 3a, the ambient temperature is varied over a range exceeding 10 °C through continual heating and cooling phases during a 90‑min test (Fig. 3b). Throughout these variations, the auxiliary laser frequency is dynamically adjusted—increasing during heating and decreasing during cooling—to shift the cavity resonance via the thermo‑optic effect, thereby maintaining the pump‑cavity detuning within the soliton existence range. This active compensation is reflected in the remarkably stable single soliton power, which fluctuates within only ±0.015 dB. The repetition rate remains tightly locked, with an Allan deviation of 6.2 × 10^-12^ at 1 s and 1.9 × 10^-13^ at 1000 s (Fig. 3c), remaining at a comparable level to that under unperturbed conditions (5.6 × 10^-^¹² at 1 s and 1.6 × 10^-13^ at 1000 s). The phase noise of the repetition rate also shows excellent consistency under multimodal locking (Fig. 3d). This minimal degradation demonstrates that the multimodal locking effectively decouples the comb’s frequency stability from ambient temperature changes.

We further subject the system to mechanical vibration tests to evaluate its performance under dynamic strain. The packaged microresonator chip is mounted on a vibration platform oscillating vertically at 25 Hz, and the vibration profile is monitored by an accelerometer placed in close proximity to the resonator (Fig. 3e). The acceleration amplitude exceeds ±4 g (Fig. 3f, 1 g equals 9.8 m s^-2^). Note that under vibration with the multimodal locking, the single-soliton power fluctuates by over 0.1 dB without collapse, yet remains below the ~ 0.25 dB threshold where soliton collapse occurs (Fig. 2g‑2, gray line). This clearly illustrates the system’s ability to tolerate disturbances far beyond the collapse threshold of an unlocked comb. The power fluctuation increases with vibration acceleration, and when the vibration stops after 90 min, the power returns to its original, undisturbed state, which further demonstrates the robustness of our system. Such power fluctuation is primarily attributed to vibration-induced variations in the fiber-to-chip coupling. Unlike the output power, the stability of the repetition rate is almost unaffected by vibration. The Allan deviation is 6.4 × 10^−12^ at 1 s and 1.9 × 10^−13^ at 1000 s, closely matching the performance under static conditions (Fig. 3g). The phase noise exhibits significant consistency, except for a minimal degradation at high offset frequency (Fig. 3h).

These experiments represent, to our knowledge, the first comprehensive environmental robustness demonstration for a low‑repetition‑rate soliton microcomb. The results validate that our multimodal locking architecture not only extends the comb’s continuous operation beyond 48 h in a quiet lab setting, but also ensures its frequency stability remains largely uncompromised under realistic environmental stressors. This dual capability of long‑term stability coupled with robustness marks a critical step toward deploying soliton microcombs in portable and field‑ready metrological systems.

### Optical frequency calibration and ranging demonstration

**Fig. 4:**
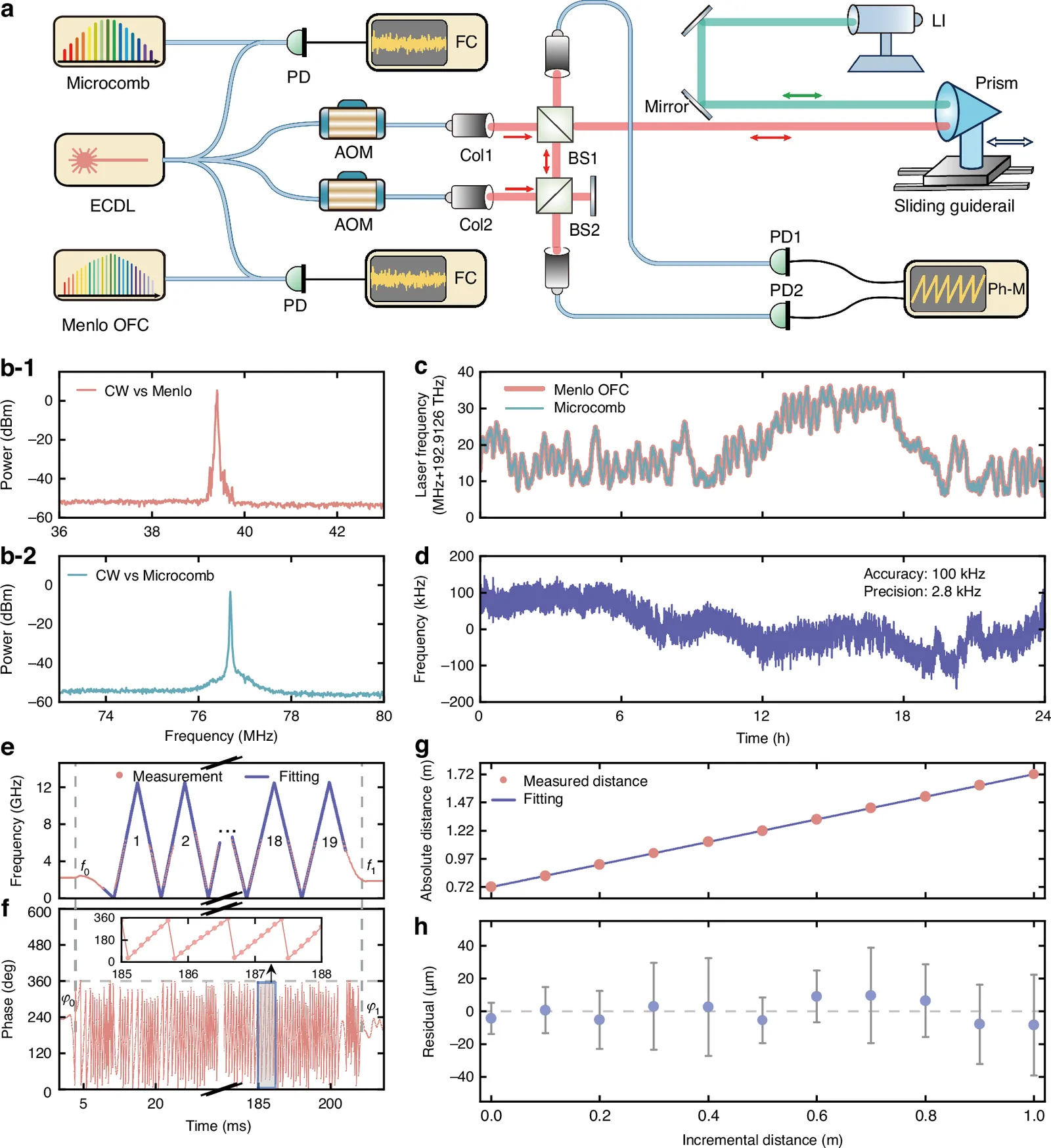
**Optical frequency calibration and frequency-swept interferometry ranging demonstration**. **a** Experimental setup. ECDL, external cavity diode laser; BS, beam splitter; Col, collimator; Ph-M, phase meter; LI, laser interferometer. **b** Beat-note spectra between the CW laser and the Menlo OFC (b-1) and our microcomb (b-2). **c** CW laser frequency traces derived from the two beat notes over 24 hours, showing excellent consistency. **d** Frequency difference between the two traces in c, remaining within ±100 kHz caused by the temperature-induced pump frequency drift and synchronization errors of FCs. **e** Recorded beat note (orange dots) and its triangular fitting (blue line) during a single laser frequency sweep. *f*_0_ and *f*_1_ denote the start and end frequencies. **f** Corresponding periodic phase during the sweep. *φ*_0_ and *φ*_1_ mark the start and end phases. **g** Comparison of the absolute distances measured by FSI (using the microcomb) and incremental distances measured by the reference LI, with linear fitting. **h** Residuals of the FSI measurements relative to the linear fit. All values lie within ±10 *μ*m (standard deviation < 30 *μ*m)

The fully determined frequencies and tunable repetition rate of the stabilized soliton microcomb enable it to serve as a precise and stable optical frequency ruler^50^. We demonstrate this through two application experiments: optical frequency calibration of an unknown continuous-wave (CW) laser and absolute distance measurement via frequency-swept interferometry (FSI). First, the microcomb serves as an absolute frequency reference to measure a CW laser (~ 1554 nm) from an external-cavity diode laser (ECDL). As shown in Fig. 4a, the CW laser is simultaneously heterodyned with our stabilized microcomb (amplified by a low-power EDFA) and a commercial Menlo OFC (referenced to a Rb clock). The beat notes are recorded in parallel by frequency counters over 24 h, with typical beat spectra shown in Fig. 4b. The microcomb-based beat exhibits comparable signal characteristics (3 dBm power, 55 dB signal-to-noise ratio, SNR) compared to the Menlo reference (-3 dBm power, 50 dB SNR). The extracted CW laser frequencies from both references show remarkable agreement over the entire measurement period (Fig. 4c, see Materials and Methods for details). Critically, the frequency difference between the two traces remains within ±100 kHz with an uncertainty of 5.2 × 10^−10^ (Fig. 4d). This corresponds to a sub‑1‑fm wavelength accuracy—nearly two orders of magnitude superior to the 10 MHz‑level accuracy of typical commercial wavelength meters^51^. The measurement precision, reflected in the Allan deviation, reaches 2.8 kHz at 1 s averaging time (Supplementary Note 8, Fig. S15b), confirming the exceptional frequency stability of our system.

To further validate the comb’s metrological performance, we conduct an FSI-based ranging experiment, making full use of the comb’s frequency calibration capability^52,53^. A heterodyne interferometer is constructed (Fig. 4a), with a commercial laser interferometer (LI) providing reference displacement measurements. During ranging, the CW laser frequency is swept over ~ 475 GHz at a scan rate of about 20 nm s^-1^. The beat note between the swept laser and the microcomb (Fig. 4e) and the corresponding interferometric phase (Fig. 4f) are recorded. Since beat signals below 1 GHz and above 10 GHz are filtered out (see Materials and Methods), the recorded beat note appears as discontinuous triangular waveforms, which can be recovered via triangular fitting. The frequency‑sweep range (Δf) and the accumulated phase shift ($\Delta \varphi$) are given by $\Delta f={f}_{0}+M{f}_{{{\rm{rep}}}}-{f}_{1}$, and $\Delta \varphi =\left({\varphi }_{0}+360N+{\varphi }_{1}\right)\cdot 2\pi /360$, respectively, with *f*_0_ (*φ*_0_) and *f*_1_ (*φ*_1_) the initial and final beat frequencies (phases), *M* (*N*) the number of frequency (phase) sweep cycles. The absolute distance *L* is then obtained by $L=c\Delta \varphi /\left(4\pi n\Delta f\right)$^54^, where *c* is the velocity of light, and n the refractive index of air (see Supplementary Note 8 for details). Figure 4g presents a comparison of measured absolute distances obtained from FSI here and incremental distances using the LI, showing excellent linearity. Figure 4h shows the fitting residuals and their standard deviation, where the residuals are derived from 10 repeated measurements of a single fixed target distance. The ranging fitting residuals are better than ±10 *μ*m, with an uncertainty of 4.33 *μ*m for *L* = 1 *m* (see Supplementary Note 8 for the uncertainty budget).

The achieved *μ*m-level ranging precision without THz-level sweep ranges^55^ and linear sweep lasers^56^, while demonstrating the viability of microcomb‑enabled FSI, is presently constrained by several systematic factors extrinsic to the microcomb itself. These include environmental disturbance, guiderail vibration-induced target drift during the relatively slow scan (~ 20 nm s^-1^), and limitations in signal processing, any of which can amplify the ranging errors. The relatively large standard deviation is mainly caused by target drift. Critically, these are not fundamental limitations of the frequency‑stabilized comb, but rather engineering challenges associated with the current proof‑of‑concept FSI setup. They can be effectively addressed through known strategies such as increasing frequency scan speed and range, implementing active vibration isolation, and employing advanced real‑time signal processing (e.g., Kalman filtering)^53,56,57^. Thus, the demonstration results of optical frequency calibration and FSI-based ranging already validate the core capability of our stabilized microcomb to serve as a precise optical ruler, while outlining a clear pathway for further performance improvement in integrated systems.

## Discussion

**Fig. 5:**
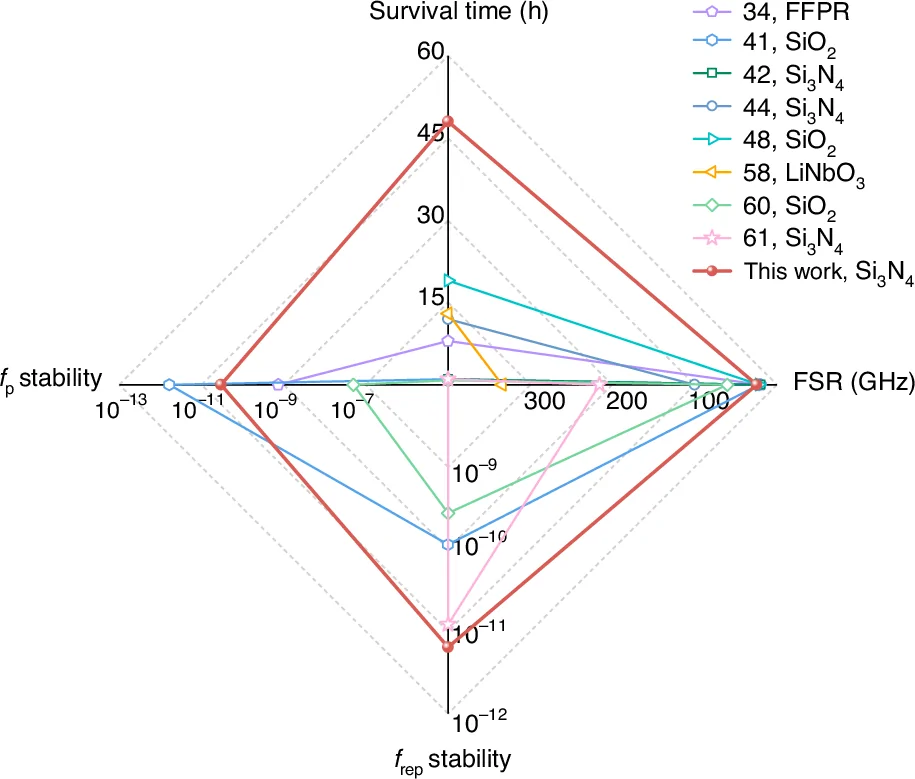
**Performance comparison of soliton microcomb stabilization schemes**. Key metrics—soliton survival time, microresonator free‑spectral range (FSR), Allan deviation of the pump frequency, and Allan deviation of the repetition rate—are plotted for several state‑of‑the‑art systems, including self‑injection‑locked combs^34^, frequency‑stabilized combs^41^, reconfigurable microcombs^61^, repetition‑rate‑locked combs^44,60^, detuning-locked combs^42,48^ and free‑running combs^58^ implemented in various platforms (SiO₂, Si_3_N_4_, LiNbO_3_, and fiber Fabry–Perot resonators), as well as the frequency-stabilized microcomb (Si_3_N_4_ microresonator) presented in this work. Here, we achieve the longest survival time (> 48 h) among low‑FSR (≤ 26.5 GHz) devices while simultaneously attaining pump‑ and repetition‑rate stabilities better than 10^-11^ at 1 s, demonstrating the unique capability of the multimodal‑locking architecture to combine long‑term robustness with frequency control

In Fig. 5, we discuss the performance of our multimodal-locked soliton microcomb compared with several state-of-the-art soliton microcombs, highlighting four key advances: extended soliton survival time, microresonator’s FSR, operation at a low microresonator FSR, and high frequency stability for both the pump laser and the repetition rate. Firstly, we achieved continuous soliton operation for over 48 h at a repetition rate of 24.96 GHz, which is a remarkable feat for a sub-26.5 GHz device. Low-repetition-rate solitons are inherently more sensitive to thermal perturbations and thus notoriously difficult to sustain. While previous demonstrations have reported long-term stability, they have predominantly relied on high-repetition-rate microresonators (often > 100 GHz) where thermal dynamics are more manageable^44,58^. Our work successfully bridges this critical gap, bringing the advantages of low-repetition-rate microcombs (direct electronic detection) into the realm of long-term reliability. Secondly, our approach achieves frequency stabilization, a feature often missing in prior works focusing solely on longevity^34,48^. By locking *f*_p_ via MTS to the Rb transition line and locking *f*_rep_ via microwave injection locking to a Rb-clock-referenced RF source, we achieve fractional frequency stabilities of 1.3 × 10^−11^ for the pump frequency and 5.6 × 10^-12^ for the repetition rate at 1 s. Compared to passive OEO with self-injection locking^59^, our scheme offers superior noise suppression at low offset frequencies and unparalleled long-term stability. These values are competitive with or surpass those reported in previous work^34,60,61^. Typically, establishing an absolute “optical frequency ruler” across the entire spectrum relies on the f‑2f self‑referencing^62^, which requires an octave‑spanning optical spectrum—a significant challenge for low‑repetition‑rate microcombs. In contrast, our multimodal locking architecture provides a simple, robust and effective approach for stabilizing both the pump frequency and the repetition rate, forming a solid foundation for metrological applications.

Note that the prominent aspect of our work lies in the conceptually versatile multimodal locking architecture, not in the specific implementation of parameter locking. This architecture exhibits cross-platform universality, which can be adapted to different materials (e.g., AlN and SiO_2_) by adjusting feedback bandwidth based on thermal response, and to various FSRs and Q‑factors by tailoring servo parameters and locking precision accordingly.

Further integration of the multimodal-locked architecture is feasible. The essential requirement of the proposed architecture is the coordinated stabilization of the pump frequency, the cavity resonance, and the repetition rate. Each of these functions can be implemented with compact photonic and electronic components. Previous work has demonstrated the integration of semiconductor lasers, on-chip amplifiers, and silicon nitride microcavities^36,63–65^. Microwave injection locking can be implemented using on-chip electro-optic modulators^66^. Furthermore, lasers, phase modulators, and photodetectors can also be heterogeneously integrated^67^. The AOM-based auxiliary-laser tuning can be replaced by an integrated in-phase/quadrature modulator^68,69^ or by an on-chip microheater^70,71^ for thermal compensation. In addition, cascaded-microresonator Vernier filters could replace the FBG to separate the soliton signal from the pump^72^. A miniature optical reference can be used to lock the pump frequency, reducing the size of the present MTS module^73,74^.

In conclusion, we have developed a robust and frequency-stabilized soliton microcomb with a low repetition rate of 24.96 GHz on the Si_3_N_4_ platform. The core of our achievement lies in the multimodal-locked architecture, which actively stabilizes the three fundamental parameters of a microcomb: the pump laser frequency, the cavity resonance frequency and thus the pump-cavity detuning, and the repetition rate. This approach not only enables record-long, collapse-free operation, but also ensures outstanding robustness against environmental perturbations. Even in extreme conditions where the temperature fluctuates by more than 10°C and the mechanical vibration acceleration exceeds ±4 g, it can still maintain single soliton operation, with repetition rate stability below 10^-11^ at 1 s, demonstrating exceptional robustness against environmental shocks. The practical utility of our stabilized microcomb is further confirmed through optical frequency calibration and high-precision FSI ranging. Our soliton microcomb agrees well with a commercial Menlo OFC within ±100 kHz over 24 h for frequency calibration, and the measured distances show residuals within ±10 *μ*m compared to a reference laser interferometer over a 1 m displacement. Our work thereby establishes a critical milestone toward the practical deployment of soliton microcombs in broad field-ready applications, transforming them from a laboratory curiosity into a reliable engine for high-precision metrology.

## Materials and methods

### Multimodal locking theory

The Lugiato-Lefever equation (LLE) describing the evolution of soliton microcombs is expressed as follows^32^:

1 $$\frac{\partial \psi }{\partial T}=-(1+i\zeta )\psi +\frac{i}{2}\frac{{\partial }^{2}\psi }{\partial {\theta }^{2}}+i|\psi {|}^{2}\psi +f$$

Here, the normalized intracavity field $\psi =A\sqrt{2g/\kappa }$, *A* is the slowly varying envelope of the intracavity field, g is the normalized Kerr nonlinear coefficient, and *κ* is the total loss rate of the resonator. The other normalized parameters are defined as: $\zeta =2({\omega }_{{{\rm{p}}}}-{\omega }_{0})/\kappa$, $\theta =\tau \sqrt{\kappa /2{D}_{2}}$,T=κt/2. With the weakly modulated pump $f={f}_{0}\cdot {e}^{i{\beta }_{{{\rm{m}}}}\sin (\Omega t)}$, we separate the real and imaginary parts of the modulated LLE and get:

2 $$\frac{\partial \phi }{\partial T}=-\zeta +{\rho }^{2}-\frac{{f}_{0}{\beta }_{{{\rm{m}}}}}{\rho }\cdot \,\sin (\Omega t-\phi )$$

where *β*_m_ is the modulation depth, and Ω is the modulation angular frequency. We define the phase difference between the modulation signal and the soliton $\varTheta =\varOmega t-\phi$, leading to

3 $$\frac{\partial \varTheta }{\partial T}=\Delta \omega +{{\mathcal{T}}}\,\sin \Theta$$

The corresponding locking range is $\delta {\omega }_{{{\rm{lock}}}}=2{{\mathcal{T}}}=2{f}_{0}{\beta }_{{{\rm{m}}}}/\rho$.

This results in the Adler equation of multimodal locking architecture with the scaled detuning $\varDelta \omega$ (including pump laser frequency, cavity resonance frequency, and modulation frequency) and coupling strength of the pump ${{\mathcal{T}}}$(see Supplementary Note 2 for derivation details). Therefore, to achieve multimodal locking, the right-hand side of the equation must be canceled out. First, the external RF frequency Ω and power (i.e., *β*_m_) need to be adjusted to achieve frequency locking (i.e., synchronization between the soliton and the modulation $\partial \varTheta /\partial T=0$). Second, to ensure long-term stability, the pump-cavity detuning must also be locked to prevent environmental fluctuations from causing the soliton to exceed the locking range and lose synchronization.

### Device information

The commercial Si_3_N_4_ photonic chip employed in our experiment is fabricated by Qaleido Photonics using the subtractive process^75^, which enables ultra-low-loss optical waveguides. The ring resonator is embedded within a SiO_2_ cladding and critically coupled to a bus waveguide. The microresonator, with a radius of 910 *μ*m, width of 2 *μ*m, and height of 810 nm, exhibits a loaded quality factor of 4.4 × 10^6^ measured using the modulation sideband method and an FSR of 24.96 GHz^76^. Moreover, the cavity features anomalous dispersion at the pump wavelength to support soliton microcomb formation.

### Repetition rate detection

The pump light of the soliton microcomb is first filtered by an FBG. The comb’s repetition rate is detected after being amplified by a low-power EDFA, and probed using an electrical spectrum analyzer (Rohde & Schwarz FSW50) and a phase noise analyzer (APPH20G). The repetition rate is approximately 25 GHz, exceeding the bandwidth of frequency counters. The repetition rate is first detected with a fast photodetector (PD), then divided by 100 to be delivered to a frequency counter (Keysight 53210A) to record its frequency fluctuation after being amplified by a low-noise amplifier.

### Experimental details of optical frequency calibration and ranging

In the optical frequency calibration demonstration, to ensure accurate beat signal acquisition by the two frequency counters and synchronization of their timestamps, both instruments are simultaneously referenced to a common rubidium clock. Data acquisition is triggered concurrently using a common trigger. The high-precision multi-channel synchronous phase meter used in the experiment is developed in-house, achieving a phase resolution of 0.77 mrad, an electronic subdivision factor of 8192, and a maximum data update rate of 10 kHz. In Fig. 4c, for the soliton microcomb, the CW laser frequency can be obtained from ${f}_{{{\rm{CW}}}}={f}_{{{\rm{p}}}}\pm \mu {f}_{{{\rm{rep}}}}\pm {f}_{{{\rm{beat}}}}$. The pump laser frequency *f*_p_ is stabilized by the MTS. The sign of ${f}_{{{\rm{beat}}}}$ can be determined by varying the CW laser frequency and observing the corresponding beat note shift. The magnitude and sign of *μ* are obtained by $\mu =\left({f}_{{{\rm{p}}}}-{f}_{{{\rm{b}}}}\right)/{f}_{{{\rm{rep}}}}$, where the ${f}_{{{\rm{b}}}}$ is the CW laser frequency roughly read out by an OSA (resolution, 0.02 nm). For the Menlo OFC, the CW laser frequency is given by ${f}_{{{\rm{CW}}}}={f}_{{{\rm{ceo}}}}\pm j{f}_{{{\rm{rep}}}}\pm {f}_{{{\rm{beat}}}}$, where ${f}_{{{\rm{ceo}}}}$ is the carrier-envelope offset frequency and *j* is the relative mode index. We initially tune the CW frequency to determine the sign of ${f}_{{{\rm{beat}}}}$, while *j* is obtained by switching the repetition rate of the Menlo OFC and observing the change of beat note.

During ranging, as shown in Fig. 4a, the output beam from the ECDL is split into three paths via a polarization-maintaining fiber coupler. The first portion is used for wavelength detection by beating with the microcomb, and the beat note is recorded by a frequency counter. The second and third portions are used to generate the reference and measurement beams. Since the phase meter requires input signals at 1 MHz ± 200 kHz, we set the driving frequencies of the two AOMs in Fig. 4a to 100 MHz and 101 MHz, respectively, to generate the target 1 MHz photodetection beat notes. The optical power after each AOM is approximately 30 *μ*W. Both beams are then separated by two beam splitters (BSs). Taking the beam from Col1 as an example, after passing through BS1, one part is combined with the beam from Col2 and detected by PD2 as the reference beam. The other part is reflected by a prism mounted on a sliding rail, and is then combined with the Col2 beam to form the measurement beam, which is detected by PD1. The signals from both photodetectors are sent to a phase meter. To enable the frequency counter to capture the beat note between the frequency-swept laser and the microcomb, the beat signal (below 12.5 GHz) detected by the fast PD is first amplified by a 10 GHz bandwidth amplifier. After passing through a 1 GHz high-pass filter, it undergoes frequency division before being transmitted to the frequency counter.

## Supplementary information

Supplementary Information for manuscript

## Data Availability

The datasets generated during and/or analyzed during the current study are available from the corresponding author on reasonable request.
