# Supplementary material for: Multimodal locking-enabled robust and day-scale low-repetition-rate soliton microcomb for high-precision metrology
Source: Light Sci Appl. 2026 Sep 7;15:370. doi: 10.1038/s41377-026-02460-w (PMC13550575; doi:10.1038/s41377-026-02460-w)
Supplement: Supplementary file 1 [file 41377_2026_2460_MOESM1_ESM.docx]

**Supplementary Information for**

**Multimodal Locking-enabled Robust and Day-scale Low-repetition-rate Soliton Microcomb for High-precision Metrology**

### Jian Tang^1,2^, Jun Yang^1,2,*^, Enqi Yan^1,2^, Guangyao Huang^1,2^, Ke Yin^3^, Donglai Tian^1,2^, Xiaoyang Lei^1,2^, Jiyuan Huang^1,2^, Mingyue Yang^1,2^, Haoran Ding^1,2^, Shuhua Yan^1,2^, Ke Wei^4^, Lingxiao Zhu^1,2^, Guochao Wang^1,2,*^, Tian Jiang^4,*^

^1^ College of Intelligence Science and Technology, National University of Defense Technology, 410073 Changsha, China

^2^ National Key Laboratory of Equipment State Sensing and Smart Support, National University of Defense Technology, Changsha, China

^3^ College of Advanced Interdisciplinary Studies, National University of Defense Technology, 410073 Changsha, China

^4^ College of Science, National University of Defense Technology, 410073 Changsha, China

These authors contributed equally: Jian Tang, Jun Yang

### Corresponding authors

^*^Correspondence to: Jun Yang ([jyang@nudt.edu.cn](mailto:jyang@nudt.edu.cn)), Guochao Wang ([wgc.19850414@163.com](mailto:wgc.19850414@163.com)), Tian Jiang ([tjiang@nudt.edu.cn](mailto:tjiang@nudt.edu.cn))

**Supplementary Note 1.** **Thermal dynamics of the microresonator**

**1.1 Thermal-induced resonance frequency shift under double pumps**

The thermo-optic effect plays a critical role in the generation and stabilization of solitons. In a soliton microcomb, the thermal sensitivity of the microresonator becomes particularly important. These resonators are typically driven at relatively high optical powers, and their characteristics undergo significant modifications due to light absorption within a small mode volume, followed by temperature variations. Altering the intracavity power modifies the microresonator temperature, which in turn shifts the mode spectrum. The impact of temperature on microresonator performance primarily arises through two processes: thermal expansion of the material and the temperature-dependent effective refractive index. The first process affects the cavity geometry and is described by the linear thermal expansion coefficient $\alpha=dR/dT/R$, where *R* is the radius of the microresonator. The second process induces changes in the effective refractive index *n* and is similarly introduced via the thermo-optic coefficient $\gamma=dn/dT/n$. In Si_3_N_4_ microresonators, both the thermo-optic coefficient and the thermal expansion coefficient are positive, making it challenging to achieve thermal equilibrium when the pump laser is red-detuned. Under these conditions, the thermo-optic effect dominates and leads to a continuous reduction in resonance frequency as the circulating power increases during the thermal stabilization process. The resonance frequency can be written as:

$$\omega_{\mu}=\frac{\mu c}{n(T)R(T)}$$

Perform a full differential on the resonance frequency to examine its change with temperature *T*:

$$\frac{d\omega_{\mu}}{dT}=\frac{\partial\omega_{\mu}}{\partial n}\frac{dn}{dT}+\frac{\partial\omega_{\mu}}{\partial R}\frac{dR}{dT}$$

From Eq. (S1), we have

$$\left\{\begin{matrix} \frac{\partial\omega_{\mu}}{\partial n}=-\frac{\mu c}{n^{2}R}=-\frac{\omega_{\mu}}{n} \\ \frac{\partial\omega_{\mu}}{\partial R}=-\frac{\mu c}{nR^{2}}=-\frac{\omega_{\mu}}{R} \end{matrix}\right.$$

The resonance frequency shift is therefore related to the temperature change by:

$$\Delta\omega_{\mu}=-\omega_{\mu}(\gamma+\alpha)\Delta T=-\omega_{\mu}a\Delta T$$

Then, the thermal-induced resonance frequency is expressed as:

$$\omega_{\mu}=\omega_{0}+\Delta\omega_{\mu}=\omega_{0}(1-a\Delta T)$$

Here, we define a thermal effect parameter $a=\gamma+\alpha$ to represent the combined influence of the two thermal effects, which is measured to be 3 GHz K^-1^ (Fig. S1).

In a microresonator, the total power coupled into the system is governed by the laser frequency, microresonator coupling efficiency *η*, pump-cavity detuning, and optical absorption losses^1^. In the auxiliary-laser heating method, both the pump and auxiliary lasers are coupled into the same resonance mode. Thus, the input heat can be expressed as:

Here, *P*_in,p_ and *P*_in,aux_ are the pump and auxiliary laser powers, respectively, *Q* is the loaded quality factor, *Q*_abs_ is the cavity quality factor associated with absorption loss, *ω*_p_ and *ω*_aux_ denote the pump and auxiliary laser frequencies, *ω*_r_ represents the thermal-induced resonance frequency, and Δ*ω* is the pumped resonance mode bandwidth, *Q* = *ω*_0_ /Δ*ω*.

The principle of energy conservation dictates that the net heat flow into the microresonator is the difference between the input and output heat:

where *C_p_* is the heat capacity and *K* is the thermal conductivity between the cavity mode volume and its surroundings. The thermal time constant is measured using the pump-probe method^2^, and two time constants, 2.8 *μ*s and 126 *μ*s, are obtained through double-exponential fitting (Fig. S2). Thus, the response time is $\tau=C_{p}/K=126\mu s$. When the microresonator is in thermal equilibrium, the thermal-induced drift is given by:

$$\Delta\omega_{\mu}=\omega_{\mu}-\omega_{0}=-\omega_{0}a\Delta T$$

where

$$\Delta T=\frac{Q}{Q_{\text{abs}}}(P_{\text{c,p}}+P_{\text{c,aux}})/K$$

**Fig. S1 Microresonator resonance frequency as a function of temperature.**

**Fig. S2 Measured relaxation velocity of the thermal effects**

**1.2 Thermal-induced pump-cavity detuning**

For a microresonator at thermal equilibrium or in the soliton regime, the thermal-induced effective pump-cavity detuning is defined by:

$$\delta\omega\left(T\right)=\omega_{0}-\omega_{\text{p}}-\Delta\omega_{\mu}=\delta\omega'-\Delta\omega_{\mu}$$

where $\delta\omega'$is the initial detuning.

**1.3 Thermal-induced intracavity power**

In the *N*-soliton regime, the intracavity power mainly consists of the pump power and soliton power^3^.

$$P\left(T\right)\approx\frac{f^{2}}{\delta\omega^{2}}+N\frac{2}{\pi}\sqrt{\frac{D_{2}}{\kappa}\delta\omega}$$

where *D*_2_ is the second-order dispersion at the pump frequency, and *κ* is the loaded linewidth.

Consequently, within the soliton regime, long-term thermal drift in the microresonator can be described by a loop: variations in the environment (such as pump power, pump laser frequency, vibration, or temperature) alter the chip temperature, thereby shifting the resonance frequency. This shift modifies the effective detuning *δω*, which consequently changes the intracavity power. This change in power, in turn, further perturbs the initial temperature variation. In the soliton regime, where the pump is red-detuned and inherently unstable, continuous optical heating combined with environmental perturbations can readily drive the effective pump-cavity detuning *δω* beyond the maximum soliton existence range (SER), consequently culminating in soliton instability or collapse. Here, the average soliton power is used as an error signal that is fed back to control the auxiliary laser frequency, thereby tuning the coupled auxiliary power *P*_c,aux_ to compensate for intracavity thermal fluctuations. The mechanism of microresonator dynamical thermal behavior is shown in Fig. S3.

**Fig. S3** Mechanism of the dynamic thermal behavior of the microresonator.

The maximum soliton existence range is^3^

$$\delta\omega_{\max}=\frac{\pi^{2}f^{2}}{8}$$

In addition, the Si_3_N_4_ platform possesses a relatively large thermo-optic coefficient, which increases its susceptibility to environmental fluctuations and thereby leads to more pronounced thermal instability.

**Supplementary Note 2. Multimodal locking theory for soliton stabilization**

The dynamic model of the soliton microcomb evolution can be expressed by the Lugiato-Lefever equation:

$$\frac{\partial\psi}{\partial T}=-(1+i\zeta)\psi+\frac{i}{2}\frac{\partial^{2}\psi}{\partial\theta^{2}}+i|\psi|^{2}\psi+f$$

Here, the normalized intracavity field $\psi=A\sqrt{2g/\kappa}$, *A* is the slowly varying envelope of the intracavity field, *g* is the normalized Kerr nonlinear coefficient, and *κ* is the total loss rate of the resonator. Other normalized parameters are defined as:$\zeta=2(\omega_{\text{p}}-\omega_{0})/\kappa$, $\theta=\tau\sqrt{\kappa/2D_{2}}\text{ }$,$T=\kappa t/2$.

With the EOM-modulated pump, the pump term *f* is expressed as $f=f_{0}\cdot e^{i\beta_{\text{m}}\sin\left(\Omega t\right)}$^4,5^, where *β*_m_ is the modulation depth, Ω is the modulation angular frequency. Considering the weak modulation *β*_m_ < 0.5, only the ±1 order terms are retained:

$$e^{i\beta_{\text{m}}\sin\left(\Omega t\right)}\approx1+i\beta_{\text{m}}\sin\left(\Omega t\right)$$

The normalized intracavity field *ψ* can be written as:

where *ρ* and *φ* are the amplitude and phase, respectively. Therefore,

Substituting Eq. (S17) into Eq. (S14), we arrive at:

Separating the real and imaginary parts of the above equation:

We define the phase difference, then

Using Eq. (S19) and (S20), we obtain:

Simplifying Eq. (S21), we obtain the Adler equation:

where $\Delta\omega=\frac{2}{\kappa}\Omega+\zeta-\rho^{2}$is the frequency detuning including pump-cavity detuning *ζ*, modulation frequency Ω, and nonlinear frequency shift *ρ*^2^; is the coupling strength of the pump, which is directly proportional to the pump amplitude *f*_0_ and modulation depth *β*_m_, and inversely proportional to the steady-state amplitude *ρ*. In the time domain, the modulation signal uses EOM-modulated pump to create an optical lattice trap within the microresonator, thereby locking the repetition rate to the external RF reference within the locking range.

To achieve long-term synchronization between the soliton and the modulation signal, the right-hand side of the equation must cancel out, satisfying $\partial\Phi/\partial T=0$, which can be achieved by adjusting the frequency and power of the modulating signal. Under this condition, the locking range can be expressed as:

We integrate Eq. (S22),

$$\tan\frac{\Theta}{2}=\frac{1}{K}+\frac{\sqrt{K^{2}-1}}{2}\tan\left(\frac{\Delta\omega\left(T-T_{0}\right)}{2}\frac{\sqrt{K^{2}-1}}{K}\right)$$

with. In the out-of-lock state, the repetition rate can be obtained by deriving Eq. :

$$\frac{\partial\Theta}{\partial T}=\Delta\omega\frac{\sqrt{K^{2}-1}}{K}$$

Fig. S4a shows the evolution of the RF spectrum with the slowly varying modulation frequency *f*_m_ during microwave injection locking. When the modulation frequency and repetition rate differ greatly, the modulation frequency, repetition frequency and their harmonics all exist in the RF spectrum. Within the locking range, the repetition rate is captured by the modulation frequency, and the free-running repetition rate and harmonics disappear. The locking range is 24.3 kHz at a modulation power of 5 dBm (modulation depth *β*_m_ = 0.08). After increasing *f*_m_, beyond the locking range, repetition rate and harmonic reappear. Fig. S4b shows the variation of locking range with modulation power. As the modulation power increases, the locking range increases rapidly. It should be noted that due to the limited detuning range *ζ* over which solitons exist, environmental fluctuations that cause detuning variations can break the synchronization and even disrupt the soliton state. Here, we set the modulation power above 5 dBm to achieve a large locking bandwidth, thereby preventing synchronization from being disrupted.

**Fig. S4 a** Evolution of the RF spectrum during injection locking at a modulation power of 5 dBm. The locking range is 24.3 kHz. **b** Dependence of the locking range on modulation power. **c** Simulation of repetition rate variation as a function of modulation frequency variation by Eq. . **d** EOM output light at a modulation depth *β*_m_ = 0.08.

For comparison, we measure the repetition rate stability under microwave injection locking alone. Environmentally induced cavity-resonance drift causes the repetition rate to leave the locking range within 1 hour (Fig. S5a). Fig. S5b shows the spectrum after loss of lock. Since microwave injection locking alone cannot achieve long-term stability, it is essential to employ pump frequency and resonance (pump-cavity detuning) locking to keep the repetition rate fluctuations within the locking range. Specifically, the pump frequency is locked via modulation transfer spectroscopy (MTS), the pump–cavity detuning is stabilized by monitoring the soliton power and tuning the auxiliary laser frequency accordingly, thereby ensuring that the repetition rate remains within the injection locking range.

**Fig. S5 a** Repetition rate under RF injection locking only. **b** Spectrum after loss of repetition-rate lock.

**Supplementary Note 3.** **Measurement of the microresonator Q factor**

**Fig. S6 a** Experimental setup for Q-factor measurement. **b** Transmission of the pumped microresonator mode. **c** Enlarged detail in **b**, with measured results shown in blue and Gaussian fitting displayed in red.

Fig. S6 shows a schematic diagram of the measurement of the Q factor of the microresonator by the modulation sideband method. First, the laser is electro-optically modulated to generate the sideband, the modulated laser is then coupled into the microcavity, and the laser frequency is scanned to cover the cavity resonance. The transmitted light intensity is detected synchronously, and the carrier and sideband signals of the resonance curve are obtained through photoelectric conversion. Finally, the full width at half height Δ*f* is measured by fitting the resonance. The Δ*f* is calculated by combining the known modulation frequency *f*_m_, and then Q factor is obtained. The loaded Q factor is 4.4×10^6^.

**Supplementary Note 4.** **MTS module for locking the 1560 nm laser**

Fig. S7 presents the modulation transfer spectroscopy (MTS) module for stabilizing the comb pump. After a 90/10 beam splitter, the 1560-nm seed laser (NKT) is amplified by an EDFA and frequency-doubled to 780 nm in a periodically poled lithium-niobate (PPLN) crystal. The 780 nm beam is split into an intense pump beam and a weaker probe beam. The pump is phase-modulated via an electro-optic modulator. Both beams are then converted into counter-propagating waves and directed through a rubidium vapor cell. The probe beam is detected by a photodiode. Its signal is fed into a lock-in amplifier, which is referenced to the phase modulation frequency. This setup extracts an error signal that crosses zero precisely at the atomic resonance center. The resulting error signal is sent to a servo box. The PID output is fed to the laser's voltage input. The laser frequency is manually brought near the error signal's zero-crossing before engaging the feedback loop. Finally, the PID parameters are optimized to achieve robust long-term stabilization.

**Fig. S7** Experimental setup of the MTS module for stabilizing the 1560 nm laser.

Fig. S8a shows the traces of the voltage applied to the pump laser's piezoelectric transducer, the corresponding saturated absorption spectrum, and MTS error signal. The beat note between the MTS-locked pump and Menlo OFC is shown in Fig. S8b, the beat note of the free-running pump varies by more than 100 MHz, whereas, for a measuring period exceeding 12 hours, the beat note variance is minimized to within 25 kHz.

**Fig. S8** Experimental results of MTS pump stabilization. **a** Voltage sweep delivered to the pump laser, corresponding saturated absorption spectrum and the MTS error signal. **b** Beat note between the MTS-locked pump and Menlo OFC.

**Supplementary Note 5.** **Deterministic generation of the single soliton**

To access a single-soliton state deterministically, we first characterize the soliton step with and without the auxiliary laser by modulating the pump laser with a 100 Hz triangular scan signal (5 V peak-to-peak). As shown in Figs. S9a and S9b, the multi-soliton step is expanded to 85.4 MHz with the auxiliary laser, which facilitated a higher probability of soliton generation and prevented the pump laser from slipping out of the soliton step.

**Fig. S9** Expansion and measurement of soliton steps. **a** Intracavity power evolution without auxiliary laser. **b** Intracavity power evolution with the auxiliary laser. **c** Statistical analysis of soliton step lengths of multiple solitons with varying pump power. The average soliton step length reaches its maximum at a pump power of 200 mW. **d** Soliton step lengths for multi- and single-soliton states at different AOM frequencies. **e** Single-soliton step length as a function of auxiliary-laser power at fixed pump power and AOM frequency.

Consistently, soliton microcombs generated via the auxiliary laser heating method evolve from multi-soliton states to single-soliton states. We further compare the soliton step lengths at different pump powers, AOM frequencies, and auxiliary laser power to identify parameters yielding a large SER conducive to soliton generation and maintenance. Therefore, we perform 150 repeated resonance scans and statistically analyzed the average soliton step lengths for both multi- and single-soliton states, as shown in Figs. S9c-e. First, we optimize the pump power for multi-soliton generation (N ≥ 6). The pump power is scanned from 120 mW to 280 mW in 5 mW steps at a fixed AOM frequency of 120 MHz and equal auxiliary laser power. The results show that as the pump power increases, the multi-soliton soliton step length shows a tendency to increase and then decrease. The maximum step width, ranging from approximately 40 MHz to 70 MHz for multi-soliton state, is achieved when the pump power is about 200 mW (Fig. S9c). Thus, the optimal pump power for the multi-soliton step is 200 mW. We then scan the AOM frequency from 115 MHz to 155 MHz with the pump and auxiliary powers fixed at 200 mW. The largest multi-soliton step is observed at an AOM frequency of approximately 120 MHz. Notably, the single-soliton state emerges within the AOM frequency range of 130-150 MHz, with the largest single-soliton step observed at 140 MHz (Fig. S9d). Furthermore, to determine the optimal auxiliary laser power for the single-soliton state, we fix the pump power and AOM frequency and scan the auxiliary power from 120 mW to 240 mW in 5 mW steps. The maximum average single-soliton step width of 9.5 MHz is achieved at an auxiliary power of 195 mW, as depicted in Fig. S9e. Therefore, the optimal parameter combination for generating single-soliton states in our experiment is 200 mW pump power, 195 mW auxiliary laser power, and 140 MHz AOM frequency.

Fig. S10a shows distinct soliton steps in the intracavity-power trace during temperature and auxiliary-laser scans. Sweeping the resonance from blue to red detuning by adjusting the microresonator temperature produces a sequence from a subcomb to multi-soliton, double-soliton and single-soliton states (Fig. S10b). Fig. S10c outlines the experimental configuration, including the resonator, pump laser, auxiliary laser, and chip temperature, for the manual generation of a single soliton. The detailed experimental procedures are shown below: Initially, the pump power, auxiliary power, and AOM driving frequency are set to 200 mW, 195 mW, and 120 MHz (conditions optimal for initiating the multi-soliton state and maintaining the single-soliton state), respectively. Simultaneously, both the pump and auxiliary laser frequencies are stabilized. Secondly, the microresonator temperature is adjusted via the TEC to induce a blue shift of the resonance frequency. At this point, both the pump and auxiliary lasers are blue-detuned relative to the resonance. The microresonator temperature is then further decreased until the pump laser is red-detuned and the auxiliary laser remains blue-detuned, continuing until a multi-soliton spectrum is observed. Thirdly, the AOM frequency is gradually increased while slightly heating the chip, which systematically reduces the soliton number. The single soliton microcomb is achieved when the AOM frequency reaches approximately 140 MHz. Accordingly, the generated single SMC is shown in Fig. S10b, showing a characteristic sech^2^ spectral envelope with a fitted pulse duration of 164 fs.

In the soliton region, the SER is proportional to the pump power^6^, expressed as $SER\propto P_{\text{in}}$. Therefore, pump power can be increased appropriately to capture the soliton more easily without impeding SMC generation.

**Fig. S10 a** Intracavity power trace during the soliton evolution. Ⅰ: subcombs; Ⅱ: multi-soliton state; Ⅲ: double-soliton state; Ⅳ: single-soliton state. **b** Spectra during soliton evolution. **c** Detailed configuration of resonance, pump laser, auxiliary laser and chip temperature during soliton evolution.

Due to the effects of rapid pump scanning and intracavity thermal effects, the SER measured via rapid laser frequency scanning is prone to compression. In our multimodal-locked architecture, the auxiliary laser serves to drive the cavity resonant frequency. To accurately measure the soliton SER, we first determine a cavity resonance frequency tuning coefficient of 0.7 MHz MHz^-1^ with respect to the auxiliary laser frequency (Fig. S11a). Therefore, the coefficient of pump-cavity detuning relative to the auxiliary laser frequency can be obtained by Eq. (S11) as 0.7 MHz MHz^-1^. Subsequently, we perform single-soliton destructive experiments by manually and slowly tuning the auxiliary laser frequency. Soliton collapse occurs when the AOM frequency is varied by ~ 65 MHz, yielding a single-soliton SER of 45.5 MHz.

To measure the relationship between the auxiliary laser frequency and the pump-cavity detuning, we combine a second probe beam with the auxiliary laser and inject them into the microcavity. The pump laser is locked via MTS. Meanwhile, the PDH technique is used to lock the probe beam to a cavity mode adjacent to the pumped mode, primarily to separate it from the auxiliary laser. We measure the pump-cavity detuning by *δω* = *f*_PDH_ − *f*_rep_, where *f*_PDH_ is the beat frequency between the probe beam and the pump laser. Fig. S11b shows a linear dependence of detuning on auxiliary laser frequency with a slope of 0.7 MHz MHz^-1^.

**Fig. S11** Cavity resonance frequency (**a**) and pump-cavity detuning (**b**) with respect to AOM frequency in a single-soliton state.

**Supplementary Note 6.** **Tuning of soliton power**

In this paper, the soliton power *P*_sol_ is monitored to feedback control the auxiliary laser frequency to lock the pump-cavity detuning *δω*, and thereby achieve long-term soliton operation. The soliton power is proportional to the input laser power *P*_in_ (that is, $\delta\omega\propto P_{\text{sol}}\propto P_{\text{in}}$). Here, we adjust the total coupled input laser power by changing the auxiliary laser frequency (the chip temperature fixed by the TEC) to balance the intracavity thermal stability and stabilize the pump-cavity detuning. Under different pump powers (the pump power is the same as the auxiliary laser power), we measure the power of different solitons. Fig. S12 shows the measured dependence of the soliton power for soliton number *N* on the input optical power. The power of *N*-soliton state is *N* times that of a single soliton. The soliton power increases almost linearly with the pump power. Furthermore, we measure the relationship between single soliton power and AOM frequency (auxiliary laser frequency), which similarly reveals a positive correlation between the two. This indicates that it is feasible to stabilize the pump-cavity detuning by adjusting the AOM frequency.

**Fig. S****12** Power-dependent *N*-soliton power.

**Fig. S13 Single-soliton power as a function of AOM frequency.**

**Supplementary Note 7.** **Power spectral density of vibration acceleration in robustness experiments**

**Fig. S14 Power spectral density of vibration acceleration. The PSD is derived from the monitored acceleration record in Fig. 3f of the main text. The acceleration noise peaks at 25 Hz, corresponding to the vibrational frequency of the platform. In addition, there are notable low-frequency and harmonic components, which are primarily caused by the actuator and mechanical structure of the vibration platform.**

**Supplementary Note 8.** **Optical frequency calibration and ranging demonstration**

To assess the stability of the Menlo OFC, we reference two OFCs to the Rb clock and perform frequency beats with both OFCs. The beat notes are captured by a frequency counter and analyzed for stability. Fig. S15a illustrates the commercial Menlo OFC’s stability, which is 1.4 × 10^-13^ and 1.1 × 10^-15^ at 1 s and 1000 s. Fig. S15b depicts the absolute frequency stability of the difference frequency shown in Fig. 4d of the main text, which is 2.8 kHz and 9.4 kHz at 1 s and 1000 s, respectively. There are two reasons for the long-term deterioration of frequency stability: First, diurnal temperature variations cause a slow drift in the rubidium atom cell, leading to a slight shift in the atomic resonance line. This drift is an inherent error of the atomic reference and is not a locking error of the soliton microcomb. Second, the beat notes in Fig. 4c are recorded by two independent frequency counters, each with a 100 ms gate time. Although synchronized via the same trigger, minute asynchronies between triggering and gating may introduce frequency errors, leading to cumulative errors in the difference frequency.

**Fig. S15** Frequency stability of the Menlo OFC and laser frequency calibration. **a** Allan deviation of Menlo OFC repetition rate. **b** Allan deviation of the difference frequency in Fig. 4d of the main text.

In the main text, the frequency and phase sweep ranges are $\Delta f=f_{0}+M\cdot f_{\text{rep}}-f_{1}$ and, respectively, where *f*_0_ and *f*_1_ are the initial and final beat frequencies, respectively, *φ*_0_ and *φ*_1_ are the initial and final phases, respectively, *M* is the number of beat sweep cycles, and *N* is the number of phase sweep cycles. We eliminate *M* and *N* by accumulating the beat frequencies and phases. Fig. S16a illustrates the accumulated frequency sweep range and corresponding phase variations obtained from the periodic beat frequencies and phase inversions of Figs. 4e and 4f in the main text. Figs. S16b and c depict details for the initial frequency *f*_0,accu_ and phase *φ*_0,accu_, and details for the final frequency *f*_1,accu_ and phase *φ*_1,accu_. Thus, the phase variation is, the laser frequency sweep range is $\Delta f=f_{\text{1,accu}}-f_{\text{0,accu}}=474.97\text{ GHz}$. The distance *L* can be obtained by$L=$ .

For FSI ranging, according to, the combined standard uncertainty *u*(*L*) is defined as

For the *u*(*n*) and *u*(Δ*φ*), we conduct experiments using the same phase meter under identical experimental conditions as in Reference [7], and the results are 4.26×10^-6^ and 0.78 *μ*m, respectively. For the *u*(Δ*f*), the frequency uncertainty of the swept-frequency laser is 7.05×10^-10^ according to Fig. 4c. Thus, the *u*(*L*) is

$$u(L)=\sqrt{(0.78\text{ }\mu m)^{2}+1.81\times10^{-11}L^{2}}$$

When *L*=1 *m*, *u*(*L*)=4.33 *μm.*

**Fig. S16 a** Phase variations and frequency sweep range derived from the phase and beat notes. **b** Details of initial phase and frequency. *f*_0,accu_ and *φ*_0,accu_ are the initial frequency and phase, respectively. **c** Details of final phase and frequency. *f*_1,accu_ and *φ*_1,accu_ are the final frequency and phase, respectively.

**References**

1.	Carmon, T., Yang, L. & Vahala, K. J. Dynamical thermal behavior and thermal self-stability of microcavities. *Optics Express* **12**, 4742-4750 (2004).

2.	Brasch, V. *et al*. Bringing short-lived dissipative Kerr soliton states in microresonators into a steady state. *Optics Express* **24**, 29312-29320 (2016).

3.	Herr, T. *et al.* Temporal solitons in optical microresonators. *Nature Photonics* **8**, 145–152 (2014).

4.	Cole, D. C. *et al.* Kerr-microresonator solitons from a chirped background. *Optica* **5**, 1304-1310 (2018).

5.	Taheri, H., Matsko, A. B. & Wiesenfeld, K. A. Dynamics of Dissipative Cavity Solitons in a Lattice Trap. *Laser & Photonics Reviews* **19**, 2500257 (2025).

6.	Lucas, E. *et al*. Detuning-dependent properties and dispersion-induced instabilities of temporal dissipative Kerr solitons in optical microresonators. *Physical Review A* **95**, 043822 (2017).

7.	Yang, J. *et al.* Microcomb-based synthetic wavelength interferometry with microwave photonic mixing. *IEEE Transactions on Instrumentation and Measurement* **74**, 7012708 (2025).
